# Self-Management Nursing Intervention for Controlling Glucose among Diabetes: A Systematic Review and Meta-Analysis

**DOI:** 10.3390/ijerph182312750

**Published:** 2021-12-03

**Authors:** Mi-Kyoung Cho, Mi Young Kim

**Affiliations:** 1 Department of Nursing Science, Chungbuk National University, 1 Chungdae-ro, Seowon-gu, Cheongju 28644, Korea; ciamkcho@gmail.com; 2 College of Nursing, Hanyang University, 222 Wangsimni-ro, Seongdong-gu, Seoul 04763, Korea

**Keywords:** diabetes, T1DM, T2DM, self-care, self-management, HbA1c, meta-analysis, systematic review

## Abstract

As the diabetic population increases, self-management of diabetes, a chronic disease, is important. Given that self-management nursing interventions using various techniques have been developed, an analysis of their importance is crucial. This study aimed to identify the overall effects of self-management nursing interventions on primary (HbA1c) and secondary (self-care, self-efficacy, fasting blood sugar level blood pressure, lipid, body mass index, waist circumference, distress, anxiety, depression, and quality of life) outcomes in diabetes. Systematic review and meta-analysis were used. The meta-analysis involved the synthesis of effect size; tests of homogeneity and heterogeneity; trim and fill plot; Egger’s regression test; and Begg’s test for assessing publication bias. The overall effect on HbA1c was −0.55, suggesting a moderate effect size, with HbA1c decreasing significantly after nursing interventions. Among the nursing interventions, the overall effect on HbA1c of nurse management programs, home visiting, and customized programs was −0.25, −0.61, and −0.65, respectively, a small or medium effect size, and was statistically significant. Healthcare professionals may encourage people with diabetes to engage in self-management of their glucose levels, such as patient-centered customized intervention. Interventions that reflect the individual’s characteristics and circumstances are effective in enabling self-management.

## 1. Introduction

The number of people with diabetes mellitus (DM) rose from 108 million in 1980 to 422 million in 2014. Prevalence has been rising more rapidly in low- and middle-income countries than in high-income countries [1]. The rapid increase in the number of people living with diabetes is a global crisis that places a huge burden on public health systems.

The health consequences of diabetes are as follows. Adults with diabetes have a two- to three-fold increased risk of heart attacks and strokes [2]. Combined with reduced blood flow, neuropathy in the feet increases the chance of foot ulcers, infection, and eventual need for limb amputation. Diabetic retinopathy is an important cause of blindness and occurs due to long-term accumulated damage to the small blood vessels in the retina. Diabetes is the cause of 2.6% of global blindness [3]. Self-management of diabetes is important because the consequences of poor blood glucose control are serious. Diabetes can be treated and its consequences avoided or delayed with continuous adaptation and management, including diet control, physical activity, medication, and regular screening and treatment for complications [1,4]. 

### Background

Self-management is a dynamic process in which individuals actively manage a chronic illness [5] and require a complete lifestyle change. It includes daily management by making good decisions related to health and life choices. The guidance and consultation of health care professionals are helpful for effective self-management. In addition, in a study of qualitative meta-synthesis on self-management of chronically ill patients, three categories of self-management processes were identified: focusing on illness needs, activating resources, and living with a chronic illness [5]. The chronic disease itself is a significant part of their life and must be managed independently. Thus, the intervention should be targeted at the person with chronic disease, including helping them manage themselves through the intervention rather than focusing on applying the intervention itself.

Therefore, it is necessary to look at the interventions that can improve self-management among various other intervention elements of diabetes. Strategies that enable good self-management may use a single or a combination of interventions to achieve the desired outcome. In addition to diet, exercise, and medication, various interventional factors include cognitive behavioral skills, goal setting, and self-monitoring, as well as suggestive, chain-like behavioral skills that associate established behaviors with new behaviors [6]. In addition to these various therapies, the duration or session of the intervention, the time of evaluation, and the study environment also vary.

Additionally, there is an increasing effort to promote self-care using coaching, as well as education and multiple media. Mediation using various media, such as telephone, in-person, and online is also increasing. When developing interventions to improve self-management, efforts are being made to promote self-care in consideration of various aspects, and interventions using various media are being developed along with the development of information technology (IT). Research on improving self-management is still in process, but it can be seen that the results as to which intervention is effective are varied.

Looking at research on meta-analysis related to diabetes, either only type 1 or type 2 diabetes has been studied [7], or when talking about genetic or constitutional aspects, national limitations were also placed [8]. In addition, studies have analyzed all diabetes-related interventions [8] and have included only online-based interventions, such as mobile apps or web-based interventions [9]; however, none have focused on self-management.

When the characteristics are homogeneously limited in this way, it is difficult to directly compare them with heterogeneous groups. Therefore, studies were limited to self-management but included those done in South Korea and overseas for other situations, and the type of diabetes included type 1 and type 2. We also tried to analyze the differences between them, including face-to-face and non-face-to-face interventions.

This study was conducted to find effective self-management interventions through a systematic literature review. Considering that the studies considered include domestic and foreign studies and various DM types, such as T1DM and T2DM, it is thought that the effect can be analyzed overall.

## 2. Materials and Methods

### 2.1. Design

This study was a systematic review and meta-analysis.

### 2.2. Eligibility Criteria and Outcome Variables

This study was performed based on the Guidelines of Systematic Reporting of Examination presented in the Preferred Reporting Items for Systematic Reviews and Meta-Analyses (PRISMA) statement [10]. To select studies, a systematic literature search was performed based on PICO-SD (population, intervention, comparison, outcome, study design). The population (P) in this study was patients with diabetes aged over 13 years, the intervention (I) was nursing interventions for promoting self-care or self-management, the comparison (C) group comprised patients receiving usual or standard care, the outcome (O) was defined as glucose or HbA1c, and the study design (SD) was limited to RCTs and quasi-experimental studies. Specific inclusion criteria included two-group comparison studies, including “glucose” or “HbA1c” as the outcome variable; intervention studies involving nurses as intervention facilitators; studies reporting the effects of nursing interventions using values, such as mean, standard deviation, and concrete sample size, which can calculate the effect size; and studies published in Korean or English. As for exclusion criteria, non-experimental studies; single group pre- and post-test studies among experimental studies; studies in which the subjects were patients with transient diabetes, such as gestational diabetes; studies to control blood sugar with fluid therapy; and dissertations or reports without peer review were excluded.

### 2.3. Search Strategy

Twelve electronic databases—Cumulative Index to Nursing and Allied Health Literature (CINAHL), Medline, PML, EMBASE, OVID, ProQuest Dissertations and Theses (PQDT), clinical key, research information sharing service (RISS), DBpia, Korean Studies Information Service System (KISS), Kyobo scholar, and E-article—were searched to identify research articles published until 31 May 2021. The search protocol was registered in the PROSPERO International Prospective Register of Systematic Reviews (registration No. CRD42021230959 available at https://www.crd.york.ac.uk/prospero/#searchadvanced, accessed on 12 January 2021). As an example, the electronic search strategy for PubMed is shown in the supplementary file. In addition, papers were manually searched based on references in the included studies for analysis, and Google Scholar, Google, and Naver search engines were used for comprehensive searches on related research topics. Keywords, such as “diabetic patient or diabetes”, “nursing intervention”, and “glucose or blood sugar”, selected according to the PICO, were identified before searching through the MeSH database. Search terms were tuned and adjusted for each database.

### 2.4. Quality Assessment

For the quality assessment of the selected papers, the checklists for RCTs and quasi-experimental studies included in the Joanna Briggs Institute of Critical Appraisal Tools [11] were used by two independent researchers (MYK and MKC). There was no difference according to the quality assessment items in the pilot test, and the item where there was a difference of opinion was the item on whether the data collector was clearly blind to the participants in the study. Through discussion, it was decided that scores should be given when the text was clearly described. The 13 items for quality assessment for RCTs comprised random assignment, allocation concealment, treatment group similarity, blinding of participants, blinding of delivering treatment, blinding of outcome assessor, similar treatment, follow-up completion, the intention-to-treat analysis, consistent method of assessing outcome measures between groups, reliability of outcome measures, appropriate statistical analysis, and appropriate trial design. The scores assigned were 0 (for “no” or “unclear”) and 1 (for “yes”), with a maximum possible total score of 13.

For the qualitative evaluation of the quasi-experimental studies, the clarity of causal effect, treatment group similarity, similar treatment, treatment group comparison, multiple scales, follow-up completion, consistent evaluation method of intergroup outcome scale, reliability of outcome measure, and appropriate statistical analysis were evaluated; the maximum possible total score was 9, with scores of 0 (“no” or “unclear”) and 1 (“yes”) being assigned.

### 2.5. Data Collection

Throughout the data collection and screening processes, all studies included in the analysis were independently reviewed by two researchers (MYK and MKC). First, the studies included in the analysis were assigned serial numbers according to bibliographic information. Second, the bibliographic information of each database was combined into one sheet, and duplicate documents were excluded. Third, while reviewing the title, abstract, and original text, based on inclusion and exclusion criteria, for excluded studies, a column was created on the serial numbered sheet to add the reason for exclusion as a note. Fourth, during independent quality assessments, memos of the reason for the exclusion were added to the names of the file assigned serial numbers. Fifth, after adjusting opinions on the exclusion and inclusion of each reviewed paper, the researchers extracted papers that met the selection criteria. Sixth, when final studies for analysis were selected, the study numbers of articles for analysis from the folder assigned serial numbers were reassigned. Seventh, the author(s), year of publication, country, number of study centers, fund, number of participants, characteristics of participants, research design, types and characteristics of interventions, contents of intervention, program facilitator, program period, program session, session time, measurement of post-test, outcome variables, and quality assessment scores of studies finally selected were extracted and recorded in the coding sheet.

### 2.6. Data Analysis

Descriptive statistics were analyzed by frequency, percentage, and mean. Statistical analyses, namely, merge of effect size, homogeneity, heterogeneity, and meta-regression method, were carried out using MIX 2.0—professional software for meta-analysis in Excel version 2.015 (BiostatXL, Mountain View, CA, USA). For analyzing effect size, the standardized mean difference was calculated for identical outcome variables, as well as 95% confidence intervals (CIs); for the weight of each effect size, the inverse of variance was used [12].

The overall effect was calculated using a random-effects model, and weight values were reset to account for the variance of each study participant and the heterogeneity between studies. The homogeneity of studies was tested through the null hypothesis of the chi-square distribution by calculating the Cochrane Q, which was the observed variance of the entire data. Moreover, the value of Higgins I2 was calculated, which is the actual variance for the observed variance of the entire data and represents the ratio of variances between studies [13]. The significance level for the Q value was 0.05. There was heterogeneity for I2 values greater than 50%. Publication bias was identified through the trim and fill method, Egger regression test, and Begg test since publication bias identification is generally recommended to validate the study results [14,15].

## 3. Results

### 3.1. Data Extraction

**Figure 1:**
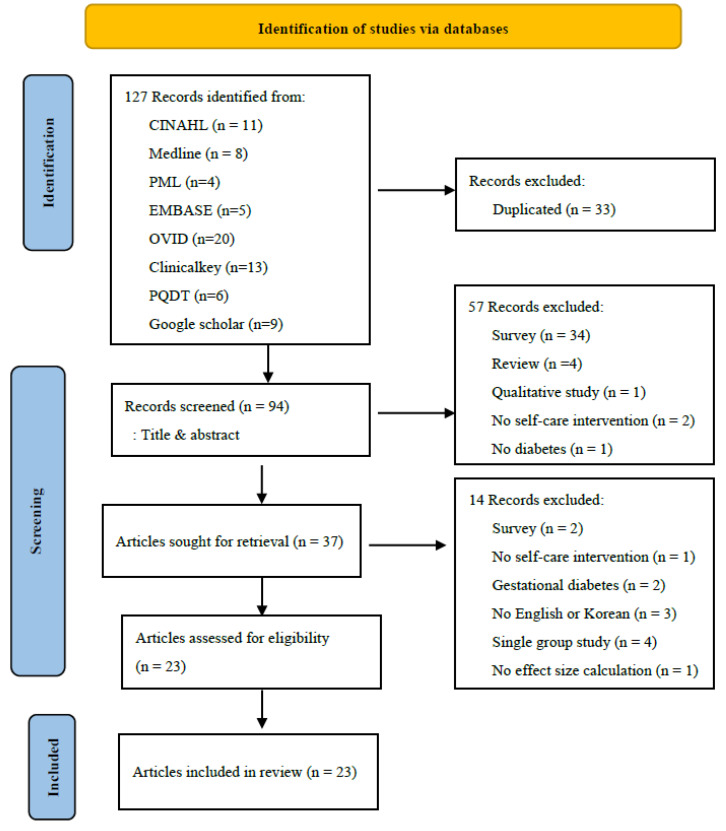
Diagram of study extraction.

The number of papers retrieved from the databases was 127. Among them, 23 articles were finally selected according to the inclusion and exclusion criteria (Figure 1). Among them, a paper by Jutterstrom et al. [16], designed using two experimental groups, was described as one study with three comparisons a-b. 

### 3.2. Characteristics of Studies

The characteristics of the studies analyzed are presented in Table 1. Fourteen studies were published after 2010, and 12 studies were published in Asia. Among all studies, nine were conducted at multiple centers and seven studies were funded. All participants in each study had diabetes, and in 17 studies the participants had T2DM. In terms of sample size, 17 studies had 50–500 participants (Table 1). 

Regarding research design, there were 12 RCTs with 1608 patients total and 11 quasi-experimental studies with 1067 patients total. The types of nursing interventions performed were nurse case management (3, study ID: 8, 9, and 23), home visiting (5, study ID: 1, 10, 18, 21, and 22), customized nursing (7, study ID: 4, 5, 7, 11, 12, 15, and 19), educational program (5, study ID: 2, 3, 13, 14, and 17), and others (3, including self-acupoint massage, cognitive behavior therapy, and nurse-led smartphone-based self-management). As for the structure of the interventions, the period of intervention was more than 12 weeks in 17 studies (study ID: 3, 5, 7, 8, 9, 11, 12, 13, 14, 15, 16, 17, 18, 19, 20, 21, and 23) and the sessions of intervention were more than 6 in 10 studies (study ID: 1, 3, 6, 7, 9, 11, 12, 13, 15, and 17), while the operating duration per session was more than 60 min in 11 studies (study ID: 2, 3, 5, 6, 7, 9, 13, 15, 17, 20, and 21).

The post-test measurement was taken immediately after the intervention in 22 studies except one (study ID 4). In outcome variables, 22 studies except one (study ID 21) measured HbA1c, while six studies assessed FBS; self-care and self-efficacy were assessed in 10 and 4 studies, respectively. As physiological variables, LDL, SBP, and DBP were measured in six studies, HDL in five studies, cholesterol and BMI in four studies, and waist circumference in two studies as an intervention effect. As for psychological variables, the intervention effect for QOL was measured in four studies and for diabetes-related emotional distress, anxiety, and depression in two studies (Table 2).

### 3.3. Methodological Quality

The mean scores of quality assessment for the 12 RCTs were 7.61 (range: 5–13) and 7.73 (range: 6–9) for the 11 quasi-experimental studies. The items of similarity of treatment groups; follow up complete and if not, adequately described and analyzed; participant analysis in the groups; and same outcome measures were described clearly in all 12 RCTs. Blinding of participants and blinding of outcome assessor were done only in two RCTs, while blinding of delivering treatment was done only in one RCT.

Among the 11 quasi-experimental studies, the items of quality assessment clarity of cause and effect, similar treatment, comparison of the treated group, multiple measures, and same outcome measure were clearly described, whereas similarity of treatment groups and appropriate statistical analysis were described clearly in five and four studies, respectively (Table 3).

### 3.4. Effect of Nursing Intervention on HbA1c

**Figure 2:**
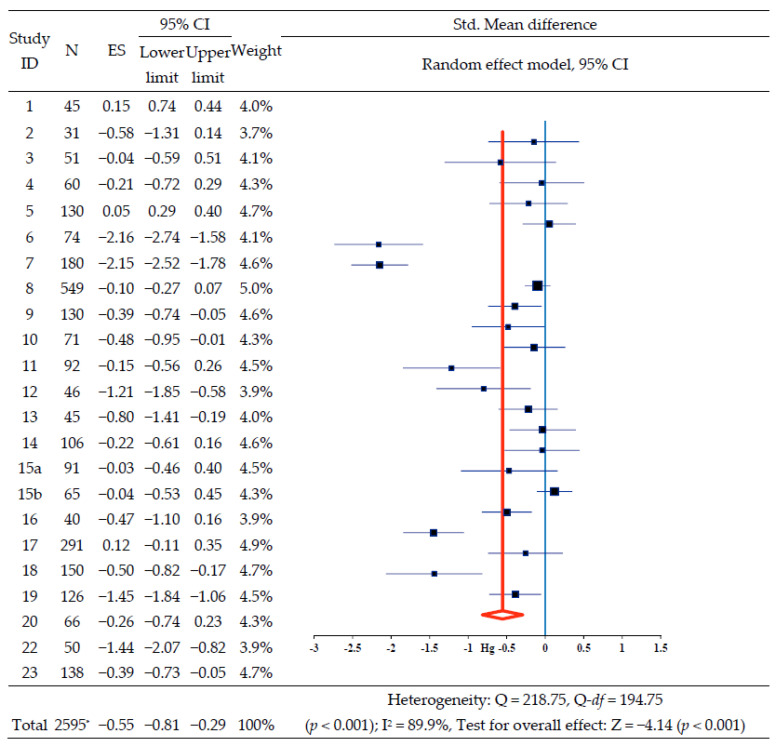
The effect of self-management nursing intervention on HbA1C. Notes. * Duplicate removal of the number of subjects Jutterstrom et al. (2016)’s study, HbA1C: glycosylated hemoglobin, ES: effect size, and CI: confidence interval.

Standardized mean differences between the two study groups in each of the 22 selected studies except one (study ID 21) were calculated using means, standard deviations, and sample sizes; the overall results are presented in a synthesis forest plot (Figure 2). The overall effect of interventions on HbA1c was −0.55 (95% confidence interval [CI]: −0.81, −0.29), which is moderate, and the HbA1c after the nursing intervention decreased significantly (Z = −4.14, *p* < 0.001). Further, the heterogeneity of the effect size was confirmed, as I^2^ was 89.9% (Q = 218.75, Q-df = 194.75, *p* < 0.001), suggesting heterogeneity of a high degree. Thus, an explorative explanation of the heterogeneity of effect sizes was found to be necessary; thereby, we conducted sub-analyses based on study characteristics, such as country, number of centers, fund, type of participants, number of participants, research design, types of intervention, period of intervention, number of intervention sessions, operating time per session, and post-test measurement (Table 4). Regardless of country, the number of study centers, funding, research design, period of intervention, intervention sessions, and operating time per session, HbA1c significantly decreased in all studies after the nursing intervention.

Contrary to the non-significant overall effect of −1.31 on HbA1c in two studies, including T1DM, the overall effect in 17 studies with T2DM was −0.47 (95% CI: −0.76, −0.18), and in three studies with diabetes (T1DM and T2DM) it was −0.52 (95% CI: −1.03, −0.02); studies were of moderate size and statistical significance.

Contrary to the non-significant overall effect on HbA1c in five studies, including fewer than 50 subjects, the overall effect in 17 studies with more than 50 subjects was −0.57 (95% CI: −0.87, −0.26), which was of moderate size and statistical significance (Z = −3.66, *p* < 0.001). Contrary to the non-significant overall effect of −0.23 with the education program and −0.96 with the other program on HbA1c, the overall effects in three studies with nurse case management program and four studies with home visiting program, including self-care education, and customized nursing, were −0.25 (95% CI: −0.47, −0.03), −0.61 (95% CI: −1.05, −0.18), and −0.65 (95% CI: −1.28, −0.02), respectively, representing a small or moderate effect size of statistical significance (Table 4).

In this study, meta-regression was additionally performed to investigate whether there was heterogeneity due to differences in study characteristics (methodological diversity) or study groups (clinical diversity). The results of the meta-regression analysis are shown in Table 5. The number of centers, funding, type of participants, number of participants, period of intervention, and the types of intervention were moderators to explain the heterogeneity of this study in meta-regression analysis. In the studies receiving funds (Z = −2.45, *p* = 0.014), of T1DM (Z = −4.20, *p* < 0.001), the number of participants more than 50 (Z = −2.76, *p* = 0.006), RCT (Z = −4.64, *p* < 0.001), and customized nursing (Z = −4.36, *p* < 0.001), HbA1c decreased significantly, whereas it increased significantly in multi-center (Z = 2.23, *p* = 0.026), intervention period more than 12 weeks (Z = 3.57, *p* < 0.001), nurse case management (Z = 3.44, *p* < 0.001), and education program studies (Z = 4.01, *p* < 0.001).

### 3.5. Effect of Nursing Intervention on Secondary Outcomes

Beyond the major outcome variable of HbA1 in the selected 23 studies, other outcome variables, such as self-care and self-efficacy as self-care related indicators; FBS, cholesterol, HDL, LDL, BP, BMI, and waist circumference as physiological indicators; and distress, anxiety, depression, QOL as psychological indicators were measured simultaneously (Table 6). The overall effects of nursing interventions on self-care (10 studies) and self-efficacy (4 studies) were significantly increased. Additionally, the overall effect on cholesterol and waist circumference was significantly increased. However, the overall effect on FBS and BMI was −0.86 (95% CI: −1.47, −0.26) and −0.64 (95% CI: −1.22, −0.07), respectively, and significantly decreased. Diabetes-related emotional distress was −0.60 (95% CI: −0.89, −0.31) and significantly decreased.

### 3.6. Publication Bias Analysis

To verify the publication bias, we used Egger’s regression test, Begg’s test, and the trim and fill method because researchers had to examine publication bias in various methods. The trim and fill method is a nonparametric (rank-based) data augmentation technique proposed by Duval and Tweedie [39], while Egger’s regression test is a more suitable linear regression method for parametric data because of the intervention effect estimates on their standard errors weighted by their inverse variance [40].

**Figure 3:**
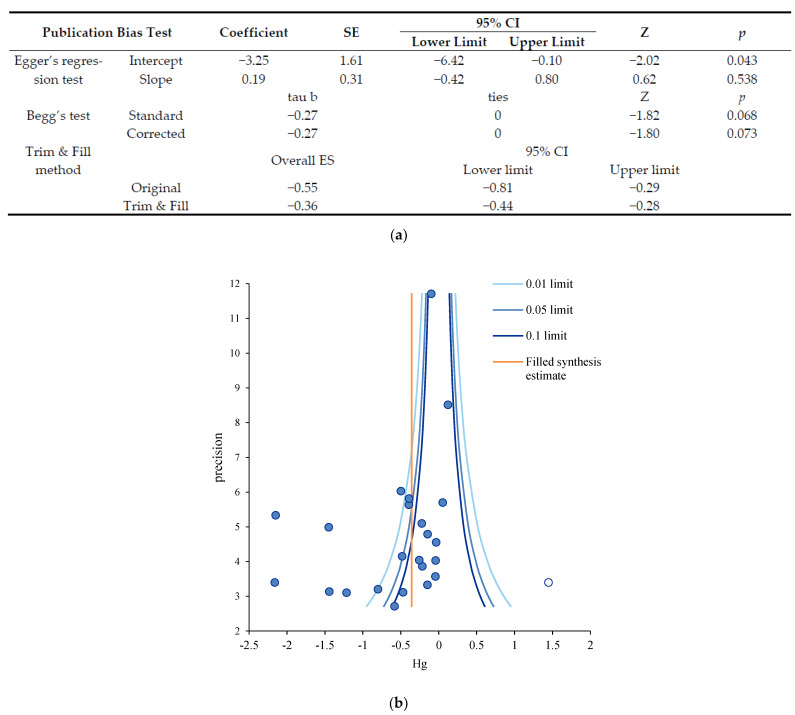
(**a**). Publication bias test of self-management nursing intervention on HbA1C. Notes. Egger’s regression test for zero intercept. Begg’s test for rank correlation; HbA1C: glycosylated hemoglobin, SE: standard error, CI: Confidence interval, and ES: effect size. (**b**). Trim and fill plot of self-management nursing intervention on HbA1C. Notes. Precision = 1/standard error, Hg = mean difference, 0.01 limit line = 99% confidence limit, 0.05 limit line = 95% confidence limit, and 0.1 limit line = 90% confidence limit.

The results of Egger’s regression test for zero intercepts showed the estimated intercept coefficient of −3.25 with a standard error of 1.61 (95% CI: −6.42, −0.10), and a *p*-value of 0.043 showed publication bias. Additionally, the Y-intercept was −3.25, which was less than 0, suggesting that the estimated intervention effect in small studies was less than that estimated in large studies. The results of Begg’s test for rank correlation showed tau b of −0.27, and ties of 0 and showed no publication bias, unlike Egger’s regression test. In the results of the trim and fill method, the original combined effect of self-management nursing intervention studies was −0.55, and the publication bias corrected overall effect size through the trim and fill method was −0.36 (95% CI: −0.44, −0.28), even if the effect size was reduced from medium to small compared to before correction. It has been shown that a self-management nursing intervention can effectively reduce HbA1c in diabetes (Figure 3a). It was shown that publication bias was corrected when one study indicated by a white circle was added to the right of the filled synthesis line of the plot (Figure 3b). Although the results of this study had some publication bias, the conclusion that could be inferred from the publication bias analysis did not appear to be at the level to say that self-managed nursing interventions for diabetes were not effective for glucose control.

## 4. Discussion

Due to the increase in the prevalence and mortality of diabetes, the need for the development and testing of diabetes intervention, medical environment, and medical technology is emerging. In this study, from numerous RCT and quasi-experimental diabetes intervention studies, the contents and effects of the interventions based on self-management were analyzed.

The results of the analysis of 23 studies finally considered are as follows. The place where the intervention was conducted included the community and medical institutions, and the participants included T1DM and T2DM patients. In one study, participants were adolescents, while in all other studies, they were adults. When examining the contents of diabetes self-management intervention by subject, it was a combination of two or more contents, such as diet, exercise, medication, lifestyle, blood glucose monitoring, and blood glucose management. The fact that interventions for diabetes have the characteristics of integrated interventions reflects the complexity of diabetes management [41]. In addition, interventions, such as cognitive behavioral therapy and complementary and alternative therapy, were also applied.

The indicator used in the included studies to measure the effect of diabetes self-management intervention was HbA1c, a representative physiological indicator of diabetes control as well as physiological indicators, such as lipids and blood pressure. In addition, there were self-management-related indicators, such as self-care and self-management efficacy, and psychosocial variables, such as depression and QoL. In a study analyzing the effects of diabetes intervention [8], because health management behavior is induced through cognitive and psychological changes, physiological indicators, such as blood glucose and HbA1c, were measured together with cognitive and psychological variables, such as self-efficacy and self-care. Although physiological measurements have the advantage of being objective, they do not reflect all aspects of health behavior. Therefore, rather than applying one method, various measures, including self-report, are necessary [42]. The development of metrics will be necessary.

Most results of related study were related to disease management, disease specificity, risk factors, and proximal factors characterized by disease management. Given that distal outcomes have been said to be related to the success of proximal outcomes and include costs related to health care [43], it is evident that socioeconomic status affects T2DM management quality in relation to healthy snack consumption [44]; ultimately, studies on distal outcomes, including costs related to health care, are also required.

The results of the analysis of HbA1c selected as the primary evaluation variable in this study are as follows. When analyzed according to the DM type of the participants, there was an effect on HbA1c reduction when only T2DM or both T1DM and T2DM were included compared to the case where only T1DM patients were participants. In the study including only T1DM, the decrease in HbA1c was not large, but it was larger when T2DM was included.

The larger effect size in T2DM was consistent with a previous meta-analysis study [44] that suggested that the type of diabetes was a decisive factor in the intervention effect. Nielsen et al. [45] pointed out that the effect of T2DM was higher than that of T1DM, and this was particularly the case with exercise intervention. It is thought that access to various aspects, such as lifestyle changes, resulted in a stable reduction of HbA1C in T2DM. The reason for this can be thought of as a difference according to the self-management intervention method and evaluation variables. If the existing generally performed self-management interventions were expanded and applied to T1DM based on the data developed for T2DM, the self-management intervention program may not be suitable for T1DM patients.

Conversely, in T1DM, because it is difficult to maintain stable blood glucose, there is a risk of hypoglycemia, and reducing glucose variability during the day is key [46]. In this respect, HbA1c—the average blood glucose for three months—does not reflect the degree of glucose variability. It is possible that it was not seen as a decrease in HbA1c. As in the previous study that mentioned the relationship between glucose variability and microvascular and macrovascular complications of diabetes [47], interest in the importance of fluctuations in blood glucose is increasing. Therefore, if the intervention is targeted at T1DM, which has high blood glucose variability, self-care intervention suitable for T1DM should be performed and the effect should be evaluated, including HbA1c as well as indicators that can reflect the range of blood glucose fluctuations when determining the endpoint.

The decrease in HbA1C was more significant when the number of participants was over 50 than when it was less than 50. Therefore, to show the significance of the intervention effect, it is suggested that the number of participants should be 50 or more. Although the characteristics of the participants’ diabetes may be similar, characteristics other than diabetes may vary, such as the type of diabetes, complications, comorbidities, age, and family circumstances. It can be seen that it is necessary to secure a sufficient number of participants to verify the significance of the intervention effect.

When the types of interventions were classified, the case management program; home visiting program, including self-care education; and customized self-management program were more effective in lowering HbA1c than the education program. In other words, the management of diabetes involves changing the entire life pattern, such as diet, exercise, and drug management, rather than adding a small change in behavior due to knowledge improvement. The application of this result might be difficult. Education should include continuous management in the forms of home visits and a customized self-management program. In some cases, home visiting was done face-to-face, but there were also cases where it was done non-face-to-face, and it was effective regardless of the method. This is consistent with studies [48] showing that reminder interventions, including all methods (e.g., telephone, text, mail, paging, interactive voice response systems, video, telephone, and programmed electronic audiovisual reminders, etc.), are effective in improving treatment adherence. In other words, it can be thought that checking and giving feedback on the situation from time to time helps maintain the changed lifestyle.

As self-management of diabetes was highly related to quality of life [49], this highlights the importance of maintaining self-management, and as indicated by the results of this study, frequent monitoring and feedback on situations were found to help maintain a changed life pattern, which supports this theory.

Looking at the program structure of most of the included studies, face-to-face education was included in the experimental group and the control group, and the interventions added to the experimental group were regular visits or regular non-face-to-face contact. Both face-to-face and non-face-to-face blood glucose levels improved in the experimental group. This explains the importance of regular contact or checking and suggests that even non-face-to-face visits can have a comparable effect to face-to-face visits. Various techniques, such as e-mail reminders, virtual education platforms, and tele-monitoring, can be used for chronic disease patients who need continuous self-management support [50]. That is, when face-to-face activities are not possible due to public health concerns, or simply to use resources effectively, the usefulness of the web or mobile-based program is expected to be high, and the possibility of application suitable for the characteristics of chronic diseases is high. It is necessary to develop and implement high-level interventions using various forms of media.

Additionally, regarding the customized self-management program, it was effective when the participant received information about the difficulties they had and how the blood glucose level or current management level was, and received customized feedback and management accordingly. This result is related to the fact that health care professionals emphasize the importance of better understanding the participant’s process of performing self-nursing and knowing where they are having difficulties [51]. For the successful management of chronically ill patients, it is necessary to develop a patient-centered self-management intervention method as it is preferred over the disease-based approach used in disease management programs [52].

The importance of coaching tailored to the target is emerging and the core of coaching is that the target is led independently. It is necessary to help the participant make their own decisions, share information, and do it independently. Given that the participant is the patient, the intervention itself was a result of showing that customized intervention considering the participant’s situation is effective.

Conversely, as a result of this study, HbA1c was not related to the country where the study was conducted, the number of centers participating in the study, whether research funding was provided, whether the study design was RCT or quasi-experimental, the duration of the intervention, the intervention session, or the duration of the intervention for each session. This result is partially consistent with that of previous studies. Regarding the intervention period, the meta-analysis of Norris, Engelgau, and Narayan [53] disagreed with the conclusion that a long-term program of more than six months was more effective in self-care and blood sugar control than a short-term program. However, in this study, the decrease in HbA1c was caused without any difference depending on the conditions and proves that the location where the study was conducted, the environment in which the study was conducted, and the external conditions related to the intervention were not significant influencing factors.

Meanwhile, the results of the analysis on secondary outcomes are as follows.

In this study, indicators related to self-management of diabetes rather than direct blood sugar levels were set as secondary outcomes and their effect sizes were analyzed. To measure the effectiveness of self-management interventions, a direct method is to use changes in blood sugar, which is a direct change, as an index. However, as one of the important aspects supporting self-management is the psychosocial aspect of behavior change to promote health and well-being [50], most studies also measured it. The secondary evaluation variables included in this study were classified into three categories as follows. These were self-management-related indicators (self-care and self-efficacy), physiological indicators (FBS, cholesterol, HDL, LDL, BP, BMI, and waist circumference), and psychological indicators (distress, anxiety, depression, and QOL).

As a result of the analysis, there was a significant increase in self-care and self-efficacy. Among physiological indicators, cholesterol, waist circumference, FBS, and BMI were significantly reduced. Among psychological indicators, diabetes-related emotional stress was significantly reduced. However, HDL, LDL, and BP among physiological indicators, and anxiety, depression, and QOL among psychological indicators, had no significant effect.

This is also the result of some inconsistencies with the study of short-term diabetes-related interventions to improve physiological indicators and QOL in a meta-analysis study by Funnell et al. [54]. Rather, because the focus was on self-management, the results for the secondary outcomes are considered to be mixed. Grey, Knafl, and McCorkle [55] reported, when evaluating effective self-management of chronic diseases, condition outcomes—outcomes for improved health; individual outcomes, including QOL and self-efficacy, and family well-being; and functioning, including familial outcomes and environmental outcomes in an extended sense, such as health care systems [55]. It can be said that it is important to consider psychosocial aspects and evaluate their effectiveness. As stated, cognitive-psychological intervention strategies should be included for effective diabetes management [54]. Self-management intervention programs, including cognitive psychosocial factors, are thought to be effective in glycemic control and psychosocial aspects.

To identify the causes of systematic heterogeneity by study, meta-regression analysis was performed by inputting study characteristics (methodological diversity) or study groups (clinical diversity). As a result, the heterogeneity was moderately explained by the number of centers, research funding, type of diabetes, duration of intervention, and type of intervention. In other words, it is necessary to consider that these characteristics may affect the heterogeneity of the results of the study. Additionally, future studies should be considered to determine to what extent core mediation and incidental mediation methods should be used in a complementary manner based on the types of diabetes addressed, specific population recruited, and duration of the interventions.

This study includes domestic and international intervention studies related to self-management of diabetes and suggests the following clinical significance. First, as this study includes domestic and foreign studies, it shows that the general aspect of diabetes can be emphasized more than differences due to genetic predisposition and environmental factors in the occurrence and management of diabetes. Second, given that this study is not limited to a particular type of diabetes, it was possible to analyze the difference in the results and it is shown that the development of an intervention method and evaluation variable suited to the type is necessary. Third, patient-centered or patient-specific interventions are effective. Fourth, maintaining self-management is important in chronic disease, and the method to achieve this purpose does not matter. Therefore, depending on the situation, face-to-face or web and application-based programs can be used to develop and apply the appropriate resources.

### Limitations

Although this study has these advantages, it has several limitations. First, there may be a publication bias because unpublished studies could not be considered. Second, all mediations have core mediation elements, but in most cases, other mediation methods are used incidentally in addition to the core mediation. Therefore, considering that the effect was analyzed with key factors, it has the disadvantage that the intervention effect can be increased.

## 5. Conclusions

This study tried to suggest future research directions for diabetes interventions by identifying components that can increase the effectiveness of diabetes self-management interventions through meta-analysis. The study characteristics and combined effect size of 23 diabetes intervention papers were calculated. The overall effect on HbA1c suggested a moderate effect size, with HbA1c decreasing significantly after nursing interventions. Among the nursing interventions, home visiting and customized programs were statistically significant. Healthcare professionals may encourage people with diabetes to engage in self-management of their glucose levels, such as patient-centered customized interventions. In this study, various management methods were introduced and utilized. Elements of inspection and management were included during self-management, such as telephone, in-person visits, and online participation. Intermediate inspection and continuous management were found to be effective even when it was done remotely, which showed the effect of non-face-to-face interventions.

The factors identified in this study can be used as basic guidelines for setting future diabetes intervention research directions and developing and operating effective diabetes intervention programs.

In an era where direct visits are difficult, further research is required to determine how much the scope and duration of non-face-to-face inspection or treatment can be extended.

## 6. Patents

This section is not mandatory but may be added if there are patents resulting from the work reported in this manuscript.

## Figures and Tables

**Table 1:** Characteristics of the included studies.

Study ID	Author	Year	Country	Center	Fund	Participants	Characteristics of Participants	Quality Score
1	Park et al. [17]	2010	Korea	1	No	T2DM, N = 45 (E: 25, C: 20)	Public health center registration, Adults > 18	5
2	Ko and Gu [18]	2004	Korea	1	No	T2DM, N = 31 (E: 17, C: 14)	Age 20–70 years, completed diabetes education	8
3	Park [19]	2021	Korea	1	No	T2DM, N = 51 (E: 26, C: 25)	Public health center registration, Adults > 18	8
4	Hyun et al. [20]	2009	Korea	1	Yes	T2DM, N = 60 (E: 30, C: 30)	Uncontrolled diabetic patients, Adults > 20, HbA1C > 6.5%, PP2 > 180 mg/dL	7
5	Sim and Hwang [21]	2013	Korea	1	No	T2DM, N = 130 (E: 65, C: 65)	OHA treatment, HbA1C > 7.0%	8
6	Amsberg et al. [22]	2009	Sweden	2	Yes	T1DM, N = 74 (E: 36, C: 38)	Age: 18–65 years, BMI < 30 kg/m^2^, HbA1c > 7.5%	8
7	Prezio et al. [23]	2013	USA	1	No	T2DM, N = 180 (E: 90, C:90)	Age 18–75 years, OHA treatment	8
8	Stuckey et al. [24]	2009	USA	2	No	T2DM, N = 549 (E: 276, C: 273)	Uncontrolled DM (HbA1c > 8.5), hypertension (BP > 140/90), hyperlipidemia (LDL > 130), Age 18–75 year	10
9	Li et al. [25]	2017	Canada	2	Yes	T2DM, N = 130 (E: 69, C: 61)	Age ≥ 18, HbA1c ≥ 8% Mean age > 55	9
10	Gurkan et al. [26]	2019	Turkey	2	No	T1DM, N = 71 (E: 35, C: 36)	Pediatric endocrinology outpatient (Adolescents: 13–17 years)	9
11	Lemelin et al. [27]	2020	Canada	1	Yes	T1DM + T2DM, N = 92 (E: 45, C: 47)	Diabetes center outpatient, Adults > 18	7
12	Thompson et al. [28]	1999	Canada	1	No	T1DM + T2DM, N = 46 (E: 23, C: 23)	Adults > 18, treated with at least one injection of insulin per day HbA1c > 8.5	13
13	Gallegos et al. [29]	2006	Mexico	2	No	T2DM, N = 45 (E: 25, C:20)	Cr < 1.5 ml/dl, Mean age > 40	6
14	Martin-payo et al. [30]	2021	Spain	4	No	T2DM, N = 106 (E: 59, C: 47)	Health care centers, Adults > 18	7
15	Jutterstrom et al. [16]	2016	Sweden	10	Yes	T2DM, N = 124 (E1: 59, E2: 33, C: 32)	Health care centers, Adults > 18	9
16	Wang et al. [31]	2020	Singapore	1	Yes	T2DM, N = 40 (E: 20, C: 20)	Tertiary acute public hospital outpatient, Adults> 21, HbA1c > 8%	5
17	Alibrahim et al. [32]	2021	Kuwait	1	No	T2DM, N = 291 (E: 150, C: 141)	Primary Health Center, Adults > 21	6
18	Chan et al. [33]	2006	Hong Kong (China)	2	No	T2DM, N = 150 (E: 75, C: 75)	Elderly > 65	7
19	Wang et al. [34]	2021	China	1	Yes	T2DM, N = 126 (E: 63, C: 63)	FBG > 7.0 mmol/L, 2-h PBG > 11.1 mmol/L, HbA1C > 6.5%, Elderly > 60	6
20	Lyu et al. [35]	2019	China	1	No	T2DM, N = 66 (E: 32, C: 34)	Elderly > 60	8
21	Chi et al. [36]	2018	China	1	No	T2DM, N = 80 (E: 40, C: 40)	Age 30–80 years	5
22	Mollaoglu and Beyazit [37]	2009	Turkey	1	No	T2DM, N = 50 (E: 25, C: 25)	Mean age > 50	7
23	Aubert et al. [38]	1998	USA	2	No	T1DM + T2DM, N = 138 (E: 71, C: 67)	Mean age > 50	9

Notes. T2DM: type 2 diabetes, T1DM: type 1 diabetes, E: experimental group, C: control group, HbA1C: glycosylated hemoglobin, LDL: low-density lipoprotein *cholesterol,* BMI: body mass index, and PP2: 2-h post-prandial glucose level.

**Table 2:** Intervention characteristics of the included studies.

Study ID	Author	Research Design	Intervention	Intervention Contents	Facilitator	Period of Intervention	Program Session	Session Time	Post-Test Measurement	Outcome Variables
1	Park et al. [17]	RCT	Home visiting	Diet, exercise, medication, and maintaining normal blood sugar range	Visiting nurse, student nurse	10 wk	10 session	not mentioned	Immediately	HbA1C, self-efficacy, self-care performance, cholesterol, HDL, and LDL
2	Ko and Gu [18]	Quasi-E	Education	Coping with Problem Situation	Nurse	4 wk	4 session	120 min	Immediately	HbA1C, self-efficacy, self- care behaviors, and coping with problematic situations
3	Park [19]	Quasi-E	Exercise, Education, and Counselling	Aquatic exercise, self-management	Researcher, aquatic exercise instructor	12 wk	24 session	80 min	Immediately	HbA1c, self- care behaviors, self-efficacy, SBP, DBP, FBS, BMI, Waist–hip ratio, and percent of body fat
4	Hyun et al. [20]	Quasi-E	Customized nursing	1:1 customized diabetes education	doctor, diabetes education nurse, nutritionist, and pharmacist	not mentioned	3 session	30 min	With delay	HbA1C, PP2, self- blood glucose, insulin injection, and lifestyle
5	Sim and Hwang [21]	Quasi-E	Customized nursing	Self-Management Education (Self-monitoring of blood glucose)	Diabetes education nurse	12 month	2 session	100 min	Immediately	Hb A1c, goal achievement on Hb A1c
6	Amsberg et al. [22]	RCT	Cognitive behavior therapy	Cognitive Behavior Therapy	Nurse	8 wk	8 session	120 min	Immediately	HbA1c, BMI, self-care behaviors, emotional distress (PAID), fear for hypoglycemia, and psychosocial factors (anxiety, depression)
7	Prezio et al. [23]	RCT	Customized nursing	Community diabetes education (CoDE) program	Community health worker (3 full-time physicians, bilingual medical assistants and clerical staff	12 months	7 session	60 min	Immediately	HbA1c, blood pressure (SBP, DBP), BMI, and lipid status (LDL, HDL, and TG)
8	Stuckey et al. [24]	RCT	Case Management	Motivational interviewing (MI) to deliver the self-management intervention	Nurse	2-year study	not mentioned	not mentioned	Immediately	HbA1c, BMI, Lipid profile, SBP, DBP, emotional distress (PAID), treatment satisfaction (DTSQ), and depression (CES-D)
9	Li et al. [25]	RCT	Nurse Case Management	Diabetes self-management education and support, monitoring and algorithm-driven treatment adjustment	Nurse	6 months	12 session	60 min	Immediately	HbA1c, diabetes distress (DDS), clinical (SBP, DBP, BMI), and behavioral and psychosocial outcome
10	Gurkan et al. [26]	Quasi-E	Home visiting	Home-based nursing intervention program	Nurse	5 wk	5 session	not mentioned	Immediately	HbA1c, self-efficacy, and diabetes behavior
11	Lemelin et al. [27]	Quasi-E	Customized nursing	Tele-home care (THC) Program	Nurse, doctor	6 months	frequently feed back	not mentioned	Immediately	HbA1c, self-efficacy, and diabetes behavior rating
12	Thompson et al. [28]	RCT	Customized nursing (insulin adjustment)	Phone contact with the nurse: advice about adjustment of insulin therapy	Diabetes nurse educator	6 months	78 session	15 min	Immediately	HbA1c, medical visits, and nursing interventions during 3-month period on THC
13	Gallegos et al. [29]	Quasi-E	Education and counselling	Educational session, counseling session	Nurse	12 months (50 weeks)	6 session	90 min	Immediately	HbA1c, diabetes self-care activities, self-care capabilities, psychological adaptation to the chronic illness, and barriers to self-care
14	Martin-payo et al. [30]	Quasi-E	Education	Educational intervention (dietary and exercise habits)	Nurse	6 months	4 session	10–20 min	Immediately	HbA1c, BMI, recommendation related to diet and exercise, motivation, competence autonomy, and social support for healthy eating and exercise
15	Jutterstrom et al. [16]	RCT	Customized nursing	E1: group intervention, E2: individual intervention	Nurse	12 months	6 session	45–90 min	Immediately	HbA1c, BMI, waist circumference, blood pressure (SBP, DBP), lipid profile (Chol, HDL, LDL, and TG)
16	Wang et al. [31]	Quasi-E	Nurse-led smartphone-based self-management	Telephone follow-up and face-to-face patient education, Care4Diabetse app sessions	Nurse	6 months	not mentioned	not mentioned	Immediately	HbA1c, Diabetes-Dependent Quality of Life (DDQoL), Revised Summary of Diabetes Self-care Activities (RSDSCA), General Self-efficacy Scale (GSS)
17	Alibrahim et al. [32]	Quasi-E	Education and self-control	DSME (diabetes self-management education)	Certified Diabetes Educator (CDE) nurses	12 months	DSME educational sessions	60 min	Immediately	HbA1c, BMI, waist circumference, and blood pressure
18	Chan et al. [33]	Quasi-E	Education and home visiting	Education (medication, diet, and exercises), telephone follow up	Nurse	3 months	6 session	30 min	Immediately	HbA1c, SBP, DBP, body weight, and PEQD (informed choice role, co-producer role, and evaluator role)
19	Wang et al. [34]	RCT	Customized nursing	Customized health education	Nurse	12 months	not mentioned	not mentioned	Immediately	Blood glucose level (HBA1c, FBS, and PP2), health management efficacy, self-rating anxiety scale (SAS), self-rating depression scale (SDS), and quality of life
20	Lyu et al. [35]	RCT	Self-acupoint massage	Routine nursing (exercise, diet, weight and caloric control, glucose monitoring, medication, and mental health assessment), self-acupoint massage,	Nurse (SEAM)	12 wk	not mentioned	120 min	Immediately	HbA1c, FBS, PP2, The Diabetes-Specific Quality of Life (QOL)
21	Chi et al. [36]	RCT	Education and home visiting	health education (knowledge of diabetes, diet and exercise, glucose management, proper insulin injection, and complications), home visits	Nurse	12 wk	5 session	60 min	Immediately	Blood glucose control of patients (FBS, PP2), self-nursing ability
22	Mollaoglu and Beyazit [37]	RCT	Education and home visiting	Nurse-initiated education program (diet, exercise, use of medication, measuring blood glucose, and urine control), home visit	Nurse	8 wk	3 session	70 min	Immediately	HbA1c, PP2, FBS, blood–urine glucose, lipid profile
23	Aubert et al. [38]	RCT	Case management	Followed written management algorithms (diet, exercise, oral agents, bedtime, and insulin)	Nurse (family physician and an endocrinologist)	12 months	Not mentioned	Not mentioned	Immediately	HbA1c, FBS, medication type and dose, body weight, blood pressure, lipid levels, patient-perceived health status, episodes of severe hypoglycemia, and emergency department and hospital admissions

Notes. RCT: randomized controlled trial, Quasi-E: quasi-experimental design, wk: week, min: minutes, HbA1C: glycosylated hemoglobin, HDL: high-density lipoprotein cholesterol, LDL: low-density lipoprotein *cholesterol,* SBP: systolic blood pressure, DBP: diastolic blood pressure, BMI: body mass index, TG: triglyceride, FBS: fasting blood sugar level, PP2: 2-h post-prandial glucose level, PAID: Problem Areas in Diabetes Questionnaire, DTSQ: diabetes treatment satisfaction questionnaire, CES-D: Center for Epidemiologic Studies Depression Scale, Chol: cholesterol, and PEQD: patients’ evaluation of the quality of diabetes care.

**Table 3:** Quality assessment of the included studies.

**Joanna Briggs Institute of Critical Appraisal Tools Checklist for Quasi-Experimental Studies**														**Total Score**
**Study ID**	**Clarity of Cause and Effect**	**Similarity of Treatment Groups**	**Similar Treatment**	**Comparison of the Treated Group**	**Multiple Measurement**	**Follow up Complete and If Not, Adequately Described and Analyzed**	**Same Way of Outcomes Measure**	**Reliable Way of Outcomes Measure**	**Appropriate Statistical Analysis**					
2	1	1	1	1	1	1	1	1	0					8
3	1	1	1	1	1	1	1	0	1					8
4	1	0	1	1	1	0	1	1	1					7
5	1	1	1	1	1	1	1	1	0					8
10	1	1	1	1	1	1	1	1	1					9
11	1	0	1	1	1	1	1	1	0					7
13	1	1	1	1	1	0	1	0	0					6
14	1	0	1	1	1	1	1	1	0					7
17	1	0	1	1	1	0	1	1	0					6
18	1	0	1	1	1	1	1	0	1					7
19	1	0	1	1	1	1	1	0	0					6
Subtotal	11	5	11	11	11	8	11	7	4					7.86
**Joanna Briggs Institute of Critical Appraisal Tools Checklist for Checklist for Randomized Controlled Trials**														**Total Score**
**Study ID**	**Random assignment**	**Allocation Concealment**	**Treatment Groups Similarity**	**Blinding of Participants**	**Blinding of Delivering Treatment**	**Blinding of Outcome Assessor**	**Similar Treatment**	**Follow up Complete and If Not, Adequately Described and Analyzed**	**Participants Analysis in the Groups**	**Same Way of Outcomes Measure**	**Reliable Way of Outcomes Measure**	**Appropriate Statistical Analysis**	**Appropriate of the Trial design**	
1	0	0	1	0	0	0	1	1	1	1	0	0	0	5
6	0	1	1	0	0	0	1	1	1	1	1	1	0	8
7	1	0	1	0	0	0	1	1	1	1	0	1	1	8
8	1	0	1	1	0	0	1	1	1	1	1	1	1	10
9	1	1	1	0	0	0	1	1	1	1	0	1	1	9
12	1	1	1	1	1	1	1	1	1	1	1	1	1	13
15	1	1	0	0	0	0	1	1	1	1	1	1	1	9
16	0	0	0	0	0	0	1	1	1	1	1	0	0	5
20	1	0	1	0	0	0	1	1	1	1	1	0	1	8
21	0	0	1	0	0	0	1	1	1	1	0	0	0	5
22	0	1	1	0	0	0	1	1	1	1	1	0	0	7
23	1	0	1	0	0	1	1	1	1	1	1	0	1	9
Subtotal	7	5	10	2	1	2	12	12	12	12	8	6	7	7.61

**Table 4:** The subgroup analysis by the study characteristics on HbA1C.

Characteristics	Subgroup	K	Study ID	N	Overall ES	95% CI		Z (*p*)	I^2^ (%)
						Lower Limit	Upper Limit		
Country	Asia	12	1, 2, 3,4, 5, 10, 16, 17, 18, 19, 20, 22	1111	−0.44	−0.75	−0.12	−2.73 (0.006)	83.5
	The others *	10	6, 7, 8, 9, 11, 12, 13, 14, 15, 23	1484	−0.68	−1.12	−0.24	−3.03 (0.002)	93.3
Center	One	13	1, 2, 3, 4, 5, 7, 11, 12, 16, 17, 19, 20, 22	1208	−0.61	−1.06	−0.16	−2.63 (0.008)	92.5
	Multi center *	9	6, 8, 9, 10, 13, 14, 15, 18, 23	1387	−0.47	−0.75	−0.19	−3.25 (0.001)	83.2
Fund	No	15	1, 2, 3, 5, 7, 8, 10, 12, 13, 14, 17, 18, 20, 22, 23	1949	−0.58	−0.66	−0.50	−14.70 (<0.001)	79.4
	Yes *	7	4, 6, 9, 11, 15, 16, 19	646	−0.60	−1.10	−0.11	−2.39 (0.017)	89.4
Participants	T1DM	2	6, 10	145	−1.31	−2.96	0.34	−1.56 (0.119)	94.9
	T2DM *	17	1, 2, 3, 4, 5, 7, 8, 9, 13, 14, 15, 16, 17, 18, 19, 20, 22	2174	−0.47	−0.76	−0.18	−3.19 (0.001)	90.2
	Both	3	11, 12, 23	276	−0.52	−1.03	−0.02	−2.03 (0.043)	74.5
Number of participants	<50	5	1, 2, 12, 13, 17	458	−0.49	−1.03	0.06	−1.76 (0.079)	81.8
	≥50 *	17	3, 4, 5, 6, 7, 8, 9, 10, 11, 14, 15, 16, 18, 19, 20, 22, 23	2137	−0.57	−0.87	−0.26	−3.66 (<.001)	91.0
Research design	RCT *	11	1, 6, 7, 8, 9, 12, 15, 19, 20, 22, 23	1528	−0.80	−1.26	−0.35	−3.47 (0.001)	93.8
	Quasi-E	11	2, 3, 4, 5, 10, 11, 13, 14, 16, 17, 18	1067	−0.24	−0.42	−0.06	−2.58 (0.010)	49.7
Type of intervention	Nurse case management	3	8, 9, 23	817	−0.25	−0.47	−0.03	−2.22 (0.026)	47.3
	Home visiting	4	1, 10, 18, 22	316	−0.61	−1.05	−0.18	−2.75 (0.006)	69.3
	Customized nursing *	7	4, 5, 7, 11, 12, 15, 19	758	−0.65	−1.28	−0.02	−2.01 (0.044)	94.2
	Education	5	2, 3, 13, 14, 17	524	−0.23	−0.56	0.10	−1.34 (0.180)	62.5
	The others	3	6, 16, 20	180	−0.96	−2.15	0.23	−1.58 (0.114)	92.6
Period of intervention	<12 weeks	6	1, 2, 4, 6, 10, 22	331	−0.83	−1.47	−0.19	−2.56 (0.011)	86.6
	≥12 weeks	16	3, 5, 7, 8, 9, 11, 12, 13, 14, 15, 16, 17, 18, 19, 20, 23	2.264	−0.46	−0.75	−0.18	−3.16 (0.002)	90.5
Intervention session	Not mentioned	5	8, 16, 19, 20, 23	919	−0.53	−1.02	−0.03	−2.09 (0.037)	89.7
	<6	7	2, 4, 5, 10, 14, 18, 22	598	−0.43	−0.74	−0.13	−2.78 (0.005)	68.7
	≥6 *	10	1, 3, 6, 7, 9, 11, 12, 13, 15, 17	1078	−0.63	−1.14	−0.11	−2.39 (0.017)	93.7
Operating time per session	Not mentioned	7	1, 8, 10, 11, 16, 19, 23	1061	−0.45	−0.82	−0.08	−2.41 (0.016)	85.1
	<60	5	4, 12, 14, 18, 22	412	−0.67	−1.09	−0.24	−3.04 (0.002)	76.0
	≥60 *	10	2, 3, 5, 6, 7, 9, 13, 15, 17, 20	1122	−0.56	−1.06	−0.06	−2.20 (0.028)	93.7
Measurement of post test	Immediately *	21	1, 2, 3, 5, 6, 7, 8, 9, 10, 11, 12, 13, 14, 15, 16, 17, 18, 19, 20, 22, 23	2535	−0.57	−0.84	−0.30	−4.11 (<.001)	90.4
	With delay	1	4	60	−0.21	−0.72	0.29	−0.83 (0.406)	-
Quality score	<7	5	1, 13, 16, 17, 19	547	−0.55	−1.24	0.15	−1.54 (0.123)	91.8
	≥7 *	17	2, 3, 4, 5, 6, 7, 8, 9, 10, 11, 12, 14, 15, 18, 20, 22, 23	2048	−0.56	−0.85	−0.26	−3.68 (<0.001)	90.0

Notes. * Including Jutterstrom et al. (2016) study, HbA1C: glycosylated hemoglobin, K: number of studies, RCT: randomized controlled trial, Quasi-E: quasi-experimental design, ES: effect size, and CI: confidence interval.

**Table 5:** Meta-regression analysis on HbA1C.

Moderators	Subgroup	Coefficients	SE	Z	*p*
Country		−0.13	0.08	−1.58	0.115
Center		0.18	0.08	2.23	0.026
Fund		−0.23	0.09	−2.45	0.014
Participants		−0.80	0.19	−4.20	<0.001
Number of participants		−0.29	0.10	−2.76	0.006
Research design		−0.38	0.08	−4.64	<0.001
Type of intervention	Nurse case management	0.30	0.09	3.44	<0.001
	Home visiting	−0.20	0.12	−1.62	0.106
	Customized nursing *	−0.39	0.09	−4.36	<0.001
	Education	0.40	0.10	4.01	<0.001
Period of intervention		0.45	0.13	3.57	<0.001
Intervention session		−0.13	0.08	−1.61	0.108
Session operating time		−0.03	0.08	−0.38	0.704
Measurement of post test		−0.18	0.26	−0.69	0.492
Quality score		−0.09	0.10	−0.86	0.391

Notes. * Including Jutterstrom et al. (2016) study, HbA1C: glycosylated hemoglobin, SE: standard error.

**Table 6:** The effect of self-management nursing intervention on secondary variables.

Variables		K	N	Overall ES	95% CI		Z (*p*)	I^2^ (%)
					Lower Limit	Upper Limit		
Self-care related variables	Self-care	10	783	1.13	0.48	1.78	3.42 (0.001)	94.2
	Self-efficacy	4	193	0.90	0.08	1.73	2.15 (0.031)	85.7
Physiologic variables	FBS	6	511	−0.86	−1.47	−0.26	−2.82 (0.005)	90.0
	Cholesterol	4	357	4.09	1.82	6.37	3.53 (<0.001)	98.6
	HDL	5	537	−0.09	−0.25	0.08	−1.01 (0.311)	0.0
	LDL	6	1086	0.16	−0.02	0.34	1.70 (0.090)	42.7
	SBP	6	761	−0.21	−0.51	0.08	−1.41 (0.157)	75.5
	DBP	6	761	−0.04	−0.29	0.20	−0.34 (0.734)	65.6
	BMI	4	472	−0.64	−1.22	−0.07	−2.19 (0.029)	81.1
	Waist circumference	2	175	0.33	0.05	0.61	2.31 (0.021)	0.0
Psychological variables	DRED	2	194	−0.60	−0.89	−0.31	−4.07 (<0.001)	0.0
	QOL	4	306	0.56	−1.42	2.55	0.56 (0.577)	98.1
	Anxiety	2	200	−1.45	−3.32	0.41	−1.52 (0.127)	96.9
	Depression	2	200	−2.11	−5.27	1.05	−1.31 (0.191)	98.6

Notes. K: number of analysis set, ES: effect size, CI: confidence interval, I^2^: heterogeneity, FBS: fasting blood sugar level, HDL: high-density lipoprotein cholesterol, LDL: low-density lipoprotein cholesterol, SBP: systolic blood pressure. DBP: diastolic blood pressure, BMI: body mass index, DRED: diabetes-related emotional distress, and QOL: quality of life.

## Data Availability

Not applicable.

## Supplementary Materials

The following are available online at www.mdpi.com/xxx/s1, search strategy and number of searches by DB.
