# Supplementary material for: Self-Management Nursing Intervention for Controlling Glucose among Diabetes: A Systematic Review and Meta-Analysis
Source: Int J Environ Res Public Health. 2021 Dec 3;18(23):12750. doi: 10.3390/ijerph182312750 (PMC8657503; doi:10.3390/ijerph182312750)
Supplement: Supplementary file 1 [file ijerph-18-12750-s001.zip › ijerph-1465513-supplementary.pdf]

Supplementary S1. Search strategy and number of searches by DB

DB		Search strategy	number of searches
Korean	RISS	당뇨병 AND (포도당 OR 혈당) AND 간호	26
	DBpia		12
	KISS		6
	Kyobo scholar		4
	E-article		3
International	Clinicalkey	diabetic patients' AND 'nursing interventions'	1
		diabets AND 'nurse case management'	5
		diabets AND 'NCM'	2
		Diabetes Research and Clinical Practice' (저널내 검색) AND 'nursing interventions' OR HbA1C	5
	EBSCOhost CINAHL Plus with FullText	diabetic patients' AND 'nursing interventions' AND (HbA1C OR Glucose OR 'blood sugar')	11
	MEDLINE (EBSCO)		8
	PML		4
	EMBASE		5
	OVID		20
	goggle Scholar		9
	PQDT	'Diabetic' AND 'glucose' AND 'nursing'	6
		Total	127

Notes. DB: data base, RISS: Research Information Sharing Service, KISS: Korean studies Information Service System, CINAHL: Cumulative Index to Nursing and Allied Health Literature, MEDLINE: National Library of Medicine's (NLM) premier bibliographic database, PQDT: ProQuest Dissertations and Theses.
